# Comparison of socio-demographic characteristics, substance, and depression among male heroin users attending therapeutic community and methadone maintenance treatment program in Nantou, Taiwan

**DOI:** 10.1186/s13011-015-0037-y

**Published:** 2015-10-27

**Authors:** Vincent Chin-Hung Chen, Meng-Huan Wu, Tsang-Yaw Lin, Yi-Feng Ho, Hsin-Yi Wang, Michael Gossop

**Affiliations:** Chang Gung Medical Foundation, Chiayi Chang Gung Memorial Hospital, 613, Chiayi County, Taiwan; Chang Gung University, 333, Tao-Yuan, Taiwan; Tsaotun Psychiatric Center, Ministry of Health and Welfare, Nan-Tou County 542, No.161, Yu-Pin Rd, Caotun Township, Nan-Tou Taiwan, R.O.C (542); King’s College London, Institute of Psychiatry, London, SE5 8AF UK

**Keywords:** Heroin, Therapeutic community, Methadone, Depression

## Abstract

**Background:**

Little is known about differences between the characteristics and psychopathological symptoms of heroin users attending TC or MMT in Asia. This study aimed to compare characteristics and prevalence of depressive disorders among male heroin users in TC and MMT program in Nantou, Taiwan.

**Methods:**

The study sample (*n* = 705) comprised male heroin users with heroin dependence recruited from the MMT program and TC program at a psychiatric center in Nantou, Taiwan between 2006 and 2014. Socio-demographic and heroin-related characteristics were obtained from self-report questionnaires. DSM-IV diagnoses of heroin dependence, major depressive disorder, and dysthymic disorder were evaluated by trained interviewers. *T*-test and chi-square test and multivariate logistic regression were performed to measure the differences on variables between samples of TC and MMT.

**Results:**

Compared to MMT, TC participants had poorer family support, higher rate of unmarried, higher rate of unemployment, earlier onset of heroin use, longer length of heroin use, and lower daily dosage of heron. MMT heroin users had higher 1-month prevalence of major depressive disorder than TC participants. We found the distribution of current major depression disorder differed between heroin users choosing different treatment models even controlling for other demographic factors, substance related factors and psychosocial factors. The underlying explanations require further investigation.

**Conclusions:**

This study found differences in the characteristics and prevalence of psychopathology. Further study to explore the effect of these differences on the outcome between MMT and TC is warranted.

## Background

Heroin is one of the world’s most widely abused illicit substance [1]. According to the report of Taiwan Surveillance System of Drug Abuse and Addiction Treatment, heroin was the most commonly abused drug, and the rate of heroin use increased from 80.9 % in 2002 to 93.8 in 2007, and then declined to 83.3 % in 2011 [2].

Besides methadone treatment, therapeutic community (TC) is also effective in reducing substance use [3] and improving mental health and quality of life [4]. However, the majority of previous studies focused on outpatients receiving methadone treatment, and less was known about heroin users in TC. Patients with heroin dependence in TC and outpatient methadone maintenance treatment (MMT) were found to have different social-demographic features [5]. The psychopathological symptom dimensions were also found to be related with the assignment to TC versus MMT [5]. Male heroin users in MMT were more severe in the ‘worthlessness-being trapped’, ‘somatic symptoms’, and ‘panic anxiety’ than TC patients [5]. Few previous studies have conducted a formal clinical assessment leading to a diagnosis of major depression diagnosis and have tended to rely upon self-reported symptoms.

Several studies reported that there were ethnic differences in symptom domains, symptom severity, and prevalence of depressive disorder among heroin users between Asians and Caucasians [6–9]. The cultural norms, conflict of values, and maladaptive perfectionism in Asia individuals have been found to be correlated with their vulnerability to depression [10, 11]. However, previous TC study samples were mainly Caucasians and we are not aware of any study exploring comorbid depressive disorders between MMT and TC model in East Asia.

According to the Narcotics Endangerment Prevention Act in Taiwan, heroin was classified as Schedule I drug. Heroin-using offenders are often granted deferred prosecution if they are willing to receive treatment in hospitals when they were caught for the first time. A positive urine test during this period of time would require them to undergo compulsory abstention in a compulsory rehabilitation institution. They may be sentenced to 6 months to 5 years in prison if they return to use heroin during 5 years after compulsory abstention. The majority of heroin users in methadone maintenance treatment (MMT) or therapeutic community (TC) at Tsaotun Psychiatric Center are under no compulsion to stay in TC or MMT program.

TC at Tsaotun Psychiatric Center is the only abstinence-oriented and drug-free residential setting in Taiwan. Heroin users with severe withdrawal symptoms are not allowed to enter TC. They need to go through acute detoxification before attending TC. Participants are generally recommended to stay in TC for 6 months to 1 year. Individuals with positive urine drug screen or disobey rules of TC when attending the community will be expelled. The TC focuses on self-help, peer-support, personal growth, and rehabilitation. Psycho-social interventions include education, counseling, motivational therapy, cognitive-behavioral therapy, and vocational training. The goal of the TC program is to enhance recognition and control of drug use cues, resolve ambivalence about change, develop effective problem-solving strategies, and prevent relapse.

The aim of the present study was to compare the socio-demographic characteristics and heroin-related characteristics among male heroin users in TC and MMT in Nantou, Taiwan. In this study, we also investigated the differences in the prevalence of depressive disorders between TC and MMT male heroin users in Nantou, Taiwan.

## Methods

### Participants and procedures

Participants in this study were recruited from the methadone maintenance treatment (MMT) program and the therapeutic community (TC) at Tsaotun Psychiatric Center in Nantou County, Central Taiwan between 2006 and 2014.

Due to the restriction of environmental management, the TC at Tsaotun Psychiatric Center only recruits male heroin users. It is known that female gender is an important risk factor for major depressive disorder [12–15]. To avoid the interference of gender when comparing the prevalence and related factors of major depressive disorder with TC individuals, female heroin users in MMT were excluded.

A total of 999 individuals entered the methadone maintenance treatment (MMT) program at Tsaotun Psychiatric Center between 2006 and 2014. After excluding individuals without the diagnosis of heroin dependence, individuals under 20 years old, individuals with illiteracy, and individuals with history of diagnosed intellectual disability, 553 MMT heroin users participated in the study.

Among the total of 264 individuals entering the therapeutic community (TC) at Tsaotun Psychiatric Center between 2006 and 2014, 173 individuals met the diagnostic criteria of heroin dependence. After excluding individuals under 20 years old, individuals with illiteracy, and individuals with history of diagnosed intellectual disability, 152 TC heroin users participated in the study. The total participating study sample is, therefore, *n* = 705.

### Measurements

All participants completed a set of self-report questionnaires administered by a trained psychiatric nurse on the first day of the MMT or TC program. Questions included age, marriage, employment, years of education, lifetime number of criminal convictions (substance related and non-substance related), age of onset of heroin use, main route of heroin administration (injection or non-injection), sharing of needles with others (yes or no, life time), sharing equipment other than needles (yes or no, life time), and lifetime suicide attempt (yes or no). Heroin in Taiwan is weighed using a traditional measuring unit: Qian. One Qian is approximately equal to 3.75 g. The frequency and dose of heroin they used per day in the past 30 days before entering treatment were asked.

The following self-administered questionnaires were approached in this study: The Chinese version of the Severity of Dependence Scale (SDS[Ch]) is a five-item scale measuring psychological dependence experiences over the past year [16]. SDS[Ch] has good validity and reliability in Taiwan and SDS[Ch] scores were positively related to greater frequency of heroin injection, heavier heroin dosage, earlier age of heroin onset and more drug-related criminal convictions [17]. The Family APGAR[Ch] score (adaptation, partnership, growth, affection, resolve) measures the level of support and communication within the family. Higher scores indicate poorer support. The Chinese version of the scale has been validated in Taiwan [18]. The LTE[Ch] (Chinese version of the List of Threatening Experiences) is a 12-item scale assessing stressful life events that has shown good psychometric properties [19]. Three items were added to the Chinese version: failing an important examination, serious problems between parents, and serious events related to children [20]. The CAGE questionnaire [21] includes four items: Have you ever felt you should Cut down on your drinking? Have people annoyed you by criticizing your drinking? Have you ever felt bad or Guilty about your drinking? Have you ever had a drink first thing in the morning to steady your nerves or to get rid of a hangover (Eye opener)? Item responses on the CAGE are scored 0 or 1, with a higher score an indication of alcohol problems. A total score of 2 or greater is considered clinically significant. Kuo et al. [22] translated the original CAGE questionnaires to the traditional Chinese version and confirmed the cross-cultural validity of the Chinese version of CAGE questionnaire (CAGE[Ch]) when measuring the alcohol drinking problems in Taiwan.

The diagnoses of heroin dependence, major depressive disorder and dysthymic disorder were based on the criteria of Diagnostic and Statistical Manual, Fourth Edition (DSM-IV). Participants were evaluated by trained interviewers using the structured diagnostic interview of Mini-International Neuropsychiatric Interview (M.I.N.I) [23], which has been validated in Taiwan [24], on the first day when they entered TC or MMT.

We estimated the 1-month prevalence of major depressive disorder, lifetime prevalence of dysthymic disorder, and lifetime prevalence of suicide attempt among study participants.

### Statistical analysis

Descriptive statistics were used to explore the characteristics of the study sample in the TC and MMT programmes. *T*-test and chi-square test were performed to determine if there were statistically significant differences on continuous and categorical variables between samples of TC and MMT. Differences were considered significant if *p* < 0.05.

Multivariate logistic regression analysis was conducted to examine if there is significant difference in the distribution of major depressive disorder in two treatment models after adjusting for demographic and substance-related variables. Differences were considered significant if the *p* value was less than 0.05. Analyses were conducted using SPSS 15.0 for Windows.

### Ethics

The study was reviewed and approved by the Institutional Review Board of the TsaoTun Psychiatric Center. All participants provided written informed consent before participation and were informed of their right to discontinue participation at any time and assurance of confidentiality.

## Results

### Demographics and characteristics of participants

The socio-demographic and substance-related characteristics and psychiatric diagnoses of the sample are summarized in Table 1.

**Table 1:** The sociodemographic and substance-related characteristics and psychiatric diagnoses of the sample

		MMT		TC			
Continuous Variables		Mean	±SD	Mean	±SD	t	*p*-value
Age (years)		36.9	7.6	35.9	6.6	1.5	0.137
Education (years)		9.8	1.9	10.4	2.3	−2.9	0.004
Life events in recent one year^b^		1.9	2.3	1.7	1.8	0.8	0.415
Substance criminal convictions^b^		2.4	1.6	2.4	2.4	−0.4	0.695
Nonsubstance criminal convictions^b^		1.3	1.4	1.2	1.4	0.7	0.495
Family APGAR[Ch] score		5.4	4.3	6.2	3.7	−2.3	0.023
Onset age of heroin use (years)		26.1	6.5	23.9	5.9	3.8	<0.001
Length of Heroin use (years)		10.8	6.0	12.1	6.2	−2.3	0.021
SDS[Ch] total score		7.0	2.7	7.2	3.1	−0.5	0.602
Dose of heroin (half qian/day)^a^		6.1	5.9	7.6	7.2	−2.2	0.031
CAGE total score		1.3	1.4	1.5	1.4	−1.4	0.155
		MMT		TC			
Categorical Variables		n	%	n	%	*χ* ^2^	*p*-value
Married		136	24.6 %	21	13.8 %	8.3	0.016
Single		275	49.7 %	83	54.6 %		
Divorced, separated, or widowed		142	25.7 %	48	31.6 %		
Employed	Yes	308	55.7 %	69	45.7 %	4.8	0.029
	No	245	44.3 %	82	54.3 %		
Route of Heroin administration	Inhalation	138	25.0 %	36	24.2 %	0.1	0.825
	Injection	413	75.0 %	113	75.8 %		
Needle sharing	Yes	59	10.7 %	9	6.0 %	3.1	0.081
	No	492	89.3 %	142	94.0 %		
Major depressive disorder (1-month)	Yes	178	32.2 %	34	22.4 %	5.5	0.019
	No	375	67.8 %	118	77.6 %		
Dysthymic disorder (lifetime)	Yes	40	7.2 %	9	5.9 %	0.3	0.573
	No	513	92.8 %	143	94.1 %		
Suicide attempt (lifetime)	Yes	147	26.6 %	37	24.3 %	1.9	0.588
	No	406	73.4 %	115	75.7 %		

^a^Half Qian = 1.875 g (number of half Qians of heroin used per day)

^b^Mann–Whitney *U* test

In socio-demographic variables, heroin users in TC had significantly more years of education (10.4 ± 2.3 vs. 9.8 ± 1.9, *p* = 0.004), higher mean Family APGAR[Ch] scores (6.2 ± 3.7 vs. 5.4 ± 4.3, *p* = 0.023), higher rate of unmarried (86.2 % vs. 75.4 %, *p* = 0.016), and higher rate of unemployment (54.3 % vs. 44.3 %, *p* = 0.029) compared to heroin users in MMT.

For substance-related variables, heroin users in TC had significantly earlier age of onset of heroin use (23.9 ± 5.9 vs. 26.1 ± 6.5, *p* < 0.001), longer length of heroin use (12.1 ± 6.2 vs. 10.8 ± 6.0, *p* = 0.021), and lower heroin daily doses (half qian/day) (7.6 ± 7.2 vs. 6.1 ± 5.9, *p* = 0.031) compared to heroin users in MMT.

For routes of administration, the rates of injection (75.8 % vs. 75 %) and inhalation (24.2 % vs. 25 %) were similar among heroin users in TC and MMT.

The mean SDS score, number of substance related criminal convictions, number of non-substance related criminal convictions, and needle sharing rate were similar among heroin users in MMT and TC.

### Prevalence of major depressive disorder and lifetime suicide attempts

Among MMT participants, the 1-month prevalence of major depressive disorder was 32.2 %. The lifetime prevalence of suicide attempts was 26.6 %. Among TC participants, the 1-month prevalence of major depressive disorder was 22.4 %. The lifetime prevalence of suicide attempt was 24.3 %.

Heroin users in MMT had a significantly higher 1-month prevalence of major depressive disorder compared to participants in TC (*p* = 0.019). MMT and TC participants had similar lifetime prevalence of suicide attempt and dysthymic disorder.

In the Multivariate logistic regression analysis, age, education, onset age of heroin use, dose of heroin, marital status, unemployment, and Family APGAR[Ch] score were included. The difference in the 1-month prevalence of major depressive disorder among participants choosing MMT and TC remained significant after adjusting above variables (Table 2).

**Table 2:** Multivariate logistic regression analysis for 1-month major depressive disorder

Variables	B	Adjusted OR	(95 % CI)	*P* Value
Treatment model (MMT/TC)	0.599	1.820	(1.127–2.940)	0.014*
Age (years)	–0.037	0.964	(0.934–0.994)	0.019*
Education (years)	0.006	1.006	(0.921–1.100)	0.889
Onset age of heroin use (years)	0.027	1.027	(0.992–1.065)	0.136
Dose of heroin (half Qian/day)^a^	–0.044	0.957	(0.926–0.989)	0.009*
Single/married	–0.356	0.701	(0.450–1.092)	0.116
Divorced, separated, or widowed/ married	–0.237	0.789	(0.487–1.277)	0.335
Unemployment	0.411	1.508	(1.067–2.131)	0.020*
Family APGAR[Ch] score	0.052	1.053	(1.012–1.097)	0.012*

*p value less than 0.05

^a^Half Qian = 1.875 g (number of half Qians of heroin used per day)

## Discussion

The present study found that heroin users entering TC and MMT in Nantou, Taiwan had different socio-demographic and substance-related characteristics. Compared to those in TC, MMT participants had higher 1-month prevalence of major depressive disorder and a higher daily dose of heroin. Heroin users in the TC had poorer family support, more years in higher education, higher rate of unmarried, higher rate of unemployment, earlier onset of heroin use, and longer length of heroin use than MMT participants.

### Socio-demographic characteristics among MMT and TC participants

TC participants in our study had higher rate of baseline unemployment, unmarried, and poorer family support than MMT participants. Our finding was consistent with a recent study in Italy [5]. The study reported that heroin users who are married or continue to work may choose a less demanding treatment such as outpatient methadone treatment, which has less impact on their job or everyday life [5]. Participants of our TC need to leave their families, give up former job, and stay in the therapeutic community for several months. The reasons stated above could make people who are married, employed, or have good relationship with families prefer MMT than TC.

Unemployment in young adulthood increased the risk of subsequent onset of heroin, even after controlling for criminal involvement [25, 26]. Unemployed heroin users have also been found to have a higher relapse rate than employed users [27]. Therefore, it is important to provide vocational education and training for people with heroin dependence, especially for TC heroin users.

The lack of social control and social support could increase the risk of developing drug disorders and relapse of heroin use [26, 28, 29]. Negative interactions with family were associated with higher major depressive disorder and depressive symptoms [30]. As a result, interventions to enhance family function and social integration for heroin users are warranted. Family members or spouses who abusing heroin or drugs also need further evaluation and treatment. The present study showed heroin users attending TC program had worse family support compared to MMT attendants. Interventions to improve family relationship and function were especially warranted for TC attendants.

### Substance-related characteristics among MMT and TC participants

The present study found that the dose of heroin used during 30 days before entering treatment was higher for MMT group than TC group. TC in this study is an abstinence-oriented and drug-free residential setting, and people who have severe heroin-withdrawal symptoms are not allowed to enter TC. Before entering TC, they need to go through acute detoxification phase and have several weeks of abstinence. Studies reported that the majority of heroin users continue abusing heroin when attending methadone maintenance program [25, 26], and this is consistent with our observation. These might be possible reasons that contribute to the heavier daily dose of heroin among MMT participants in the present study.

A previous study found that heroin users in TC had a significantly greater number of previous treatments and a longer history of heroin use than those in the MMT [5]. Our study had similar finding and this could be due to the fact that patients at their first treatment or with a short history of heroin prefer MMT to TC, because MMT is less likely to influence their everyday life [5].

### Comorbid major depressive disorder among MMT and TC participants

The 1-month prevalence of major depressive disorder among TC and MMT heroin users in this study appears to be higher than that in Taiwan general population (5.2 %) [27]. However, the different definitions of major depressive disorder and different demographic characteristics among studies make any direct comparison difficult.

The prevalence rate of major depressive disorder among MMT participants in our study was within the range of previous studies in United States, Australia, and Spain (ranging from 16 to 54 %) [13–15, 28, 29]. Heroin users with major depressive disorder were reported to have higher suicide rate, higher risk of drug overdose, poor compliance with treatment, higher relapse rate, and poor prognostic outcomes compared to individuals without major depressive disorder [15, 30–33]. Therefore, interventions for relieving depressive symptoms for heroin users in TC and MMT are warranted.

Compared to males, female heroin users were reported to have 2–3 fold higher prevalence of depression [13, 15, 34, 35] and most previous studies included both genders. Female heroin users were not recruited in this study, and this could be expected to influence the observed prevalence rate of depression.

Compared to TC patients in the present study, MMT heroin users had higher 1-month prevalence of major depressive disorder. We found the distribution of major depression disorder differed between heroin users choosing different treatment models even after controlling for other demographic factors, substance related factors and psychosocial factors. The reasons for this require more studies to detect underlying factors.

### Suicide attempt among MMT and TC participants

TC and MMT participants in our study had similar lifetime prevalence of suicide attempt and both were higher than that among general population [27, 36], and this finding is consistent with previous studies in other countries. The prevalence of lifetime suicide attempts among heroin users in our study was within the range of previous studies in Australia [37] and India [38, 39].

Studies reported that depressive symptoms, injection drug use, sexual or physical abuse histories, female gender, self-harm histories, and poor relationship with family were associated with suicide attempt among heroin dependent patients [37, 39, 40]. Therefore, heroin users with comorbid major depressive disorder and above risk factors need further suicide risk assessment and intervention.

### Limitations and strengths

Our study had a number of limitations. Gender differences in the prevalence and related factors of depressive disorder are important [12–15]. Due to the restriction of the environmental management, our TC only recruited male heroin users. In this study, we aimed to compare the prevalence of depressive disorder between TC and MMT heroin users. To avoid the interference of gender, female heroin users in MMT were excluded. The results in this study are only applicable to male heroin users. Also, the sample was recruited from one psychiatric center in Taiwan and the selection of these patients was not random. As a result, caution is needed in interpreting these results, since there may have selection bias in this study. In addition, the main objective for this study was a cross-sectional descriptive study focusing on the differences of major depressive disorder prevalence, socio-demographic and substance-related characteristics between male heroin users in TC and MMT. The study data cannot be used to draw causal conclusions.

However, the study also has certain important strengths. Recent studies have noted ethnic differences in depressive disorder [6–9] and the particular vulnerability to depression among Asia people [10, 11]. The present study was the first to compare the characteristics and comorbid depressive disorders among TC and MMT Asia individuals with heroin dependence. Also, there is a lack of major depression diagnosis by clinical interview rather than self-reported symptoms in previous studies. In this study, the DSM-IV diagnoses of heroin dependence, major depressive disorder, and dysthymic disorder were assessed by trained interviewers using structured and validated diagnostic interview.

## Conclusion

The present study found that heroin users from TC and MMT in Nantou, Taiwan had different socio-demographic and substance-related characteristics. The distribution of current major depression disorder differed between heroin users choosing different treatment models even controlling other demographic factors, substance related factors and psychosocial factors. Given the high prevalence of current major depressive disorder, active interventions for relieving depressive disorders for heroin users in TC and MMT are warranted. The influence of different characteristics on treatment outcome between TC and MMT warrants further follow-up studies.

## Abbreviations

TC: Therapeutic community
MMT: Methadone maintenance treatment
HIV/AIDS: Human immunodeficiency virus infection and acquired immune deficiency syndrome
USA: The United States of America
SDS[Ch]: The Chinese version of the Severity of Dependence Scale
LTE[Ch]: The Chinese version of the List of Threatening Experiences
DSM-IV: Diagnostic and Statistical Manual, Fourth Edition
